# PReS-FINAL-2111: Cytomegalovirus, Epstein Barr virus and Varicella-Zoster virus infections in children with juvenile idiopathic arthritis treated with biologics

**DOI:** 10.1186/1546-0096-11-S2-P123

**Published:** 2013-12-05

**Authors:** V Remy Piccolo, S Sotou-Bere, A Woerner, R Mouy, B Florkin, B Bader-Meunier, P Quartier

**Affiliations:** 1 Paediatric Rheumatology, Hospital Necker, Paris, France

## Introduction

Varicella-Zoster (VZV) virus, Cytomegalovirus (CMV) and Epstein Barr virus (EBV) infections may have a severe course in patients on immunosuppressive treatments. Data in Juvenile Idiopathic Arthritis (JIA) patients on biologics are still limited.

## Objectives

To assess the outcome of JIA children with VZV, CMV or EBV infection.

## Methods

We conducted a retrospective analysis of the notes of JIA patients followed between 2006 and 2013 in the French National reference centre for Juvenile Arthritis who developed CMV, EBV or VZV infection while on biologic treatment.

## Results

Ten 3 to 16-year-old children were included for VZV (n = 8), CMV (n = 2) or EBV (n = 1) primoinfection while on etanercept (n = 1), adalimumab (n = 2), anakinra (n = 2) or canakinumab (n = 6). Seven patients had concomitant non-steroidal anti-inflammatory (NSAID) treatment, 7 low dose predniso(lo)ne and 4 methotrexate. The 8 patients with VZV infection developed typical skin disease and marked systemic symptoms in some cases, however there was no visceral involvement. Nsaids and steroids were interrupted in all cases. Biologic therapy was interrupted in 7 cases for 5 to 150 days. One patient was treated with acyclovir IV and 3 with valacyclovir for 5 to 15 days. The 2 patients who developed primary CMV infection disclosed marked increase of transaminases and moderate increase of gamma glutamyl transferase for 3 weeks; one patient remained asymptomatic and the other one had mild digestive symptoms. Both were on canakinumab and the following injection was postponed. The patient with primary EBV infection developed a rash with fever, hepatomegaly and splenomegaly that resolved after 2 months while NSAID and biologic treatment had been interrupted and corticosteroid treatment prescribed in order to control disease activity. No patient developed macrophage activation syndrome and the 10 patients had a favorable outcome without sequellae.

## Conclusion

In JIA children on biologic, VZV, CMV and EBV infections may result in marked systemic symptoms in some cases, however patients outcome was favorable in this small series while NSAID treatment was systematically stopped, anti-viral therapy prescribed in some VZV infections and biologic therapy continued or only transiently interrupted.

## Disclosure of interest

V. Remy Piccolo: None declared., S. Sotou-Bere: None declared., A. Woerner Grant/Research Support from: Novartis, Consultant for: Chugai-Roche, R. Mouy Grant/Research Support from: Abbvie, Chugai-Roche, Novartis, Pfizer, Consultant for: Abbott-Abbvie, Novartis, Pfizer, Servier, BMS, Sobi, Speakers Bureau: Chugai-Roche, Novartis, Pfizer, B. Florkin: None declared., B. Bader-Meunier Grant/Research Support from: Abbvie, Chugai-Roche, Novartis, Pfizer, Consultant for: Abbott-Abbvie, Novartis, Pfizer, Servier, BMS, Sobi, Speakers Bureau: Chugai-Roche, Novartis, Pfizer, P. Quartier Grant/Research Support from: Abbvie, Chugai-Roche, Novartis, Pfizer, Consultant for: Abbott-Abbvie, Novartis, Pfizer, Servier, BMS, Sobi, Speakers Bureau: Chugai-Roche, Novartis, Pfizer.

